# P-2125. Evaluation of MucorGenius® Mucorales Real-Time PCR Assay with Formalin-Fixed Paraffin-Embedded Tissue Specimens

**DOI:** 10.1093/ofid/ofae631.2281

**Published:** 2025-01-29

**Authors:** Anuradha Ganesan, Erica Sercy, M Leigh Carson, Faraz Shaikh, Tiffany Levine, Wesley Campbell, Katrin Mende, John L Kiley, Ralf Bialek, David Tribble, Brian Wickes

**Affiliations:** Infectious Disease Clinical Research Program, USUHS; Henry M. Jackson Foundation for the Advancement of Military Medicine Inc, Bethesda, Maryland; Infectious Disease Clinical Research Program, Department of Preventive Medicine and Biostatistics, Uniformed Services University of the Health Sciences; Henry M. Jackson Foundation for the Advancement of Military Medicine, Inc., Washington, District of Columbia; Infectious Disease Clinical Research Program, Department of Preventive Medicine and Biostatistics, Uniformed Services University of the Health Sciences, Bethesda, MD, USA, Bethesda, MD; Census, Rockville, Maryland; Landstuhl Regional Medical Center, Landstuhl, Rheinland-Pfalz, Germany; Walter Reed National Military Medical Center, Bethesda, Maryland; Infectious Disease Clincial Research Program, JBSA Ft Sam Houston, Texas; BAMC, San Antonio, Texas; LADR Labor Dr. Kramer & Kollegen, Geesthacht, Schleswig-Holstein, Germany; Infectious Disease Clinical Research Program, Department of Preventive Medicine and Biostatistics, Uniformed Services University of the Health Sciences, Bethesda, MD, USA, Bethesda, MD; UT Health San Antonio, San Antonio, Texas

## Abstract

**Background:**

Early identification of mucormycetes in trauma-related invasive fungal wound infections (IFI) improves outcomes. A commercially available, multiplex, real-time Mucorales assay has the potential to provide results within 3 hours, hence we examined the performance of this assay.

**Figure:**
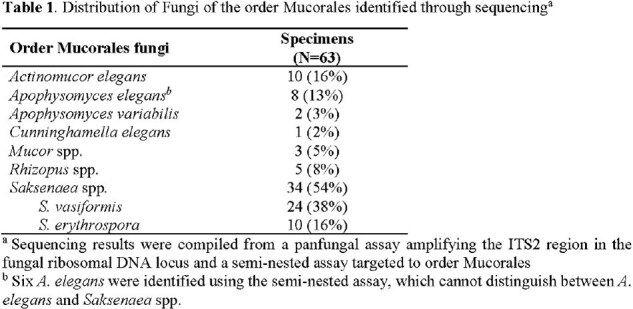

**Methods:**

Formalin-fixed specimens from combat injured that were positive for fungus on histopathology and mucormycetes on sequencing were examined using the PN-700 MucorGenius® assay (PathoNostics B.V, Maastricht, The Netherlands). The assay targets a conservative pan-mucormycetes sequence of the genera *Rhizopus*, *Mucor*, *Lichtheimia*, *Cunninghamella*, and *Rhizomucor.* For this analysis, we used a BioRad CFX96 Real-Time PCR Detection System with a positive detection limit of quantitative cycles (Cq) ≤ 30. Control specimens negative for fungus on histopathology and culture were obtained from trauma and non-trauma patients (N=2). MucorGenius® assay and sequencing results were compared.

**Figure:**
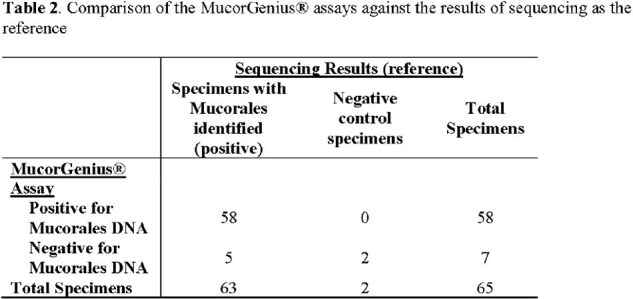

**Results:**

For this analysis, 31 patients contributed 63 specimens. Fungi of the genus *Apophysomyces* or *Saksenaea* were identified in 70% (Table 1). Using sequencing as the reference, Mucorales fungal DNA was detected in 58 specimens with the MucorGenius® assay with 5 false-negative findings (sequencing positive for *Apophysomyces elegans* [N=3], *Actinomucor elegans* [N=1], and *Mucor* spp. [N=1]), resulting in 92% sensitivity (95% confidence interval [CI]: 82-97%). There were no false positives (100% specificity; 95% CI: 16-100%; Table 2).

**Conclusion:**

To our knowledge, this is the first assessment of the MucorGenius® assay with fixed tissue specimens in trauma-related IFI. When compared with sequencing in those with histopathologically proven fungal infections, the MucorGenius® assay was sensitive and specific. The assay also successfully amplified DNA of three genera that were not targeted by the panel kit (i.e., *Apophysomyces*, *Actinomucor* and *Saksenaea*). The potential for fast results with the MucorGenius® assay offers promise for rapid diagnosis of IFI. Further assessment of performance should be conducted using fresh and fixed specimens with suspicion of trauma-related IFI.

**Disclosures:**

Ralf Bialek, n/a, InfectoPharm: Advisor/Consultant|InfectoPharm: Honoraria|InfectoPharm: podcasts|Sanofi-Adventis Deutschland GmbH: Advisor/Consultant|Sanofi-Adventis Deutschland GmbH: Honoraria|Sanofi-Adventis Deutschland GmbH: podcasts David Tribble, MD, DrPH, AstraZeneca: The IDCRP and HJF were funded to conduct an unrelated phase III COVID-19 monoclonal antibody immunoprophylaxis trial as part of US Govt COVID Response

